# Tissue culture tools for selenium hyperaccumulator *Neptunia amplexicaulis* for development in phytoextraction

**DOI:** 10.1007/s13659-022-00351-2

**Published:** 2022-08-05

**Authors:** Billy O’Donohue, Jayeni Hiti-Bandaralage, Madeleine Gleeson, Chris O’Brien, Maggie-Anne Harvey, Antony van der Ent, Katherine Pinto Irish, Neena Mitter, Alice Hayward

**Affiliations:** 1 Centre for Horticultural Science, Queensland Alliance for Agriculture and Food Innovation, The University of Queensland, Brisbane, QLD Australia; 2 Centre for Mined Land Rehabilitation, Sustainable Minerals Institute, The University of Queensland, Brisbane, QLD Australia

**Keywords:** Micropropagation, Hyperaccumulation, Phytoextraction, Selenium, Tissue culture, *Neptunia amplexicaulis*

## Abstract

*Neptunia amplexicaulis* is an herbaceous legume endemic to the Richmond area in central Queensland, Australia and is one of the strongest known Selenium hyperaccumulators on earth, showing significant potential to be utilised in Se phytoextraction applications. Here a protocol was established for in vitro micropropagation of Se hyperaccumulator *N. amplexicaulis* using nodal segments from in vitro-germinated seedlings. Shoot multiplication was achieved on Murashige and Skoog (MS) basal media supplemented with various concentrations of 6-Benzylaminopurine (BA) (1.0, 2.0, 3.0 mg L^−1^) alone or in combination with low levels of Naphthaleneacetic acid (NAA) (0.1, 0.2, 0.3 mg L^−1^), with 2.0 mg L^−1^ BA + 0.2 mg L^−1^ NAA found to be most effective. Elongated shoots were rooted in vitro using NAA, with highest root induction rate of 30% observed at 0.2 mg L^−1^ NAA. About 95% of the in vitro rooted shoots survived acclimatization. Clonally propagated plantlets were dosed with selenate/selenite solution and assessed for Se tissue concentrations using Inductively Coupled Plasma Atomic Emission Spectroscopy (ICP-AES) and found to retain their ability to hyperaccumulate. The protocol developed for this study has potential to be optimised for generating clonal plants of *N. amplexicaulis* for use in research and phytoextraction industry applications.

## Introduction

Hyperaccumulators are plants that possess the ability to uptake and accumulate incredibly high concentrations of metal(loid)s within their above-ground biomass without displaying signs of elemental toxicity [1]. It has been proposed that hyperaccumulating plants could be valuable for phytoextraction, which includes both phytomining and phytoremediation, approaching broad issues of land degradation, production limitations and resource depletion associated with many modern agricultural, mining, and industry practices [2, 3].

**Fig. 1:**
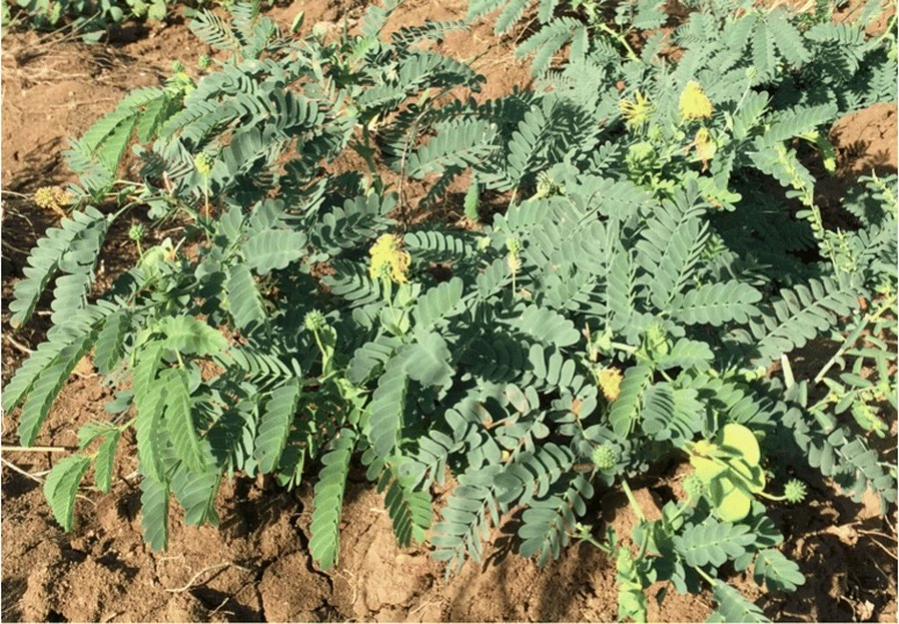
*Neptunia amplexicaulis* established in its natural habitat near Richmond in Central Queensland, Australia

*Neptunia amplexicaulis* (Selenium Weed) is a herbaceous legume endemic to the Richmond area of central Queensland, Australia, and is one of the strongest known Se hyperaccumulators on earth. When residing on the natural seleniferous soils of the Richmond area (Fig. 1) [4], Se has been recorded up to 4334 mg kg^−1^ dry weight (DW) in the plant’s aerial tissues [5, 6]. Se accumulating plants represent the main entry of Se into the food chain. The presence of these plants in agricultural or residential areas can cause cases of Selenosis in livestock and humans. Se present in the biomass of *N. amplexicaulis* is mainly in the form of organic compounds such as methyl-selenocysteine and seleno-methionine, which are of pharmacological value for diet supplements due to their high bioavailability [7]. Thus theoretically *N. amplexicaulis* could be utilised as a Se ‘crop’, deriving these bioavailable compounds from Se rich soils while simultaneously remediating the site of Se toxicity. This potential is further benefitted by the high per annum production of *N. amplexicaulis* biomass and ability to prolifically regenerate from taproots remaining in the soil [5]. As seleniferous soils are globally widespread, occurring in parts of India, China, the US, Ireland, and Australia, application of phytoextraction could be widely valuable. Such soils occur naturally through the weathering of Se rich parent material, sea spray and volcanic eruptions or result from anthropogenic activity including mineral processing, coal combustion and the production of glass and insecticides [8].

Phytoextraction is a relatively novel potential application for hyperaccumulators with various obstacles still in need of address, including the high degree of intraspecies variation in accumulation characteristics and access to plant stock. Metal accumulation has been shown to vary considerably, up to ten-fold even between individuals of the same species within the same population; additionally, many hyperaccumulating species have restricted and often threatened plant populations [5, 9–11]. In other industries such as floriculture and forestry, which historically experience similar obstacles, micropropagation has been shown to offer unique advantages and opportunities [12–14]. Micropropagation permits clonal propagation of selected individuals through the sterile in vitro culturing and manipulation of plant organs and cells, giving rise to genetically identical progeny from the mother plant [13, 15]. Effective micropropagation has been achieved for a variety of Fabaceae species as well as a few hyperaccumulating species [16–19]. Specifically, the technique of nodal culture has proven to be a reliable and productive method of in vitro micropropagation.

Nodal culture involves the promotion of axillary bud proliferation through exposure to exogenous Plant growth regulators (PGRs), mainly hormones, and manipulation of culture conditions. Propagules from elongated buds can then be sub-cultured and undergo a root induction phase, producing viable intact plantlets. Nodal culture as a direct organogenesis method offers potential for multiplication of plant propagules with reduced risk of eliciting somaclonal variation in micropropagated offspring compared to indirect organogenesis methods [20]. Nodal culture has been reported for Fabaceae plant species including *Clitoria ternatea* [17], *Desmodium gangeticum* [21] and for a Brassicaceae Se hyperaccumulating species, *Stanleya pinnata* [22]. Achieving an effective nodal culture micropropagation protocol for individuals of *N. amplexicaulis* displaying desirable accumulation and biomass production traits could provide the phytoextraction industry and research landscape with superior, reliable, and genetically consistent plant stocks.

## Materials and methods

### Plant material and surface sterilisation

*Neptunia amplexicaulis* seeds were collected from 1 year-old plants maintained by the Sustainable Minerals Institute (SMI) at The University of Queensland grown in Se-dosed natural sandy soil under temperature-controlled glasshouse conditions. Ancestry of the glasshouse grown plants is from wild seeds collected around Richmond, Central Queensland in 2018 (− 20.648359, 143.098375). Seed germination was promoted through a pre-treatment of puncturing the seed coat with a scalpel and submerging in a petri dish of distilled water for 24 h. Pre-treated seeds were sterilised by first washing twice with hand soap, washing under running water for 45 min, washing with 70% ethanol for 3 min, rinsing with sterilised water 4–6 times, washing with 2–5% bleach plus 2–3 drops of tween-20 then again rinsing 5–8 times with sterilised water.

### Media preparation and culture initiation

Plant nutrient media was prepared using Murashige and Skoog Basal salts (Austratech Pty Ltd) and Murashige and Skoog Vitamin Powder (Sigma-Aldrich Australia) supplemented with 20 g L^−1^sucrose and solidified with 7 g L^−1^ agar, final pH adjusted to 5.7 ± 0.05 using 1 M HCl and/or NaOH. For hormone treatments, media was added with specific concentration of hormones (Sigma-Aldrich Australia) before adjusting pH. Media was sterilised by autoclaving at 121 °C for 20 min. Pre-treated and surface sterilised seeds were transferred to 120 mL tubes containing 25 mL MS basal media plus vitamins for in vitro germination. Surface sterilised shoots were dissected into discrete nodes and transferred to 120 mL tubes with the same media for initiation. Percentage of contamination and shoot length was recorded after 1 month of culture. All cultures were incubated in a growth room at 25 ± 1 °C with a 16 h photoperiod at 100% relative humidity.

### Seed germination and growth media optimisation

To assess optimal media for seed germination and growth, 15 seeds were subjected to each treatment. Seeds were sterilised and sown in tubes containing 25 mL of full strength MS, half strength MS or MS media with 40 mg kg^−1^ Se. Selenium was added to media before pH balancing in the form of sodium selenate (Na_2_SeO_4_), sodium selenite (NaSeO_3_) or an equal combination of both to achieve 40 mg kg^−1^ Se (selected as it corresponds to the mid-higher range of the soil Se concentration pertaining to the site of seed collection [6]. Seed germination rates and seedling vigour was assessed between trials to identify best media for seed germination, growth, and optimal production of nodes for future experiments.

### Shoot multiplication and in vitro rooting

Nodal segments were taken from in vitro grown 4 weeks-old seedlings. The preliminary trial was conducted with MS alone or MS supplemented with 20 mg kg^−1^ Se delivered in the form of Na_2_SeO_4_, NaSeO_3_ or an equal combination of both to achieve 20 mg kg^−1^ soil Se. This was done to determine the effect low to medium levels of Se would have on nodal health and shooting performance. Additional shoot multiplication trials were performed by subculturing nodal segments onto media containing either MS alone or MS supplemented with 1.0–3.0 mg L^−1^ 6-benzylaminopurine (BA) alone or in combination with 1-naphthaleneacetic acid (NAA) at a ratio of 10:1 BA to NAA. After 4 weeks of culture, nodes were assessed for number and length of shoots per node. For rooting, elongated shoots (over 10 mm in height) were dissected from node and transferred into 120 mm tubes containing half strength MS media supplemented with 0.2, 0.6 or 1.0 mg L^−1^ NAA, submerging the bottom 2–3 mm of the basal end of the shoot in media. After 4 weeks shoots were observed for signs of root induction and root growth parameters were recorded. To ensure genomic lines of rooted shoots could be traced back to mother plant material, nodes in each culture were taken from a single seedling and likewise each rooting culture contained shoots taken from a single shoot multiplication culture. This ensured the final rooted shoots within a single culture were of the same genotype.

### Acclimatization of rooted shoots

In vitro rooted shoots were removed from culture, roots washed thoroughly with warm water to remove any excess media and soaked in 1 g L^−1^ systemic fungicide powder (Fongarid) for 8 min. Shoots were then planted out in 150 mL square pots containing with soil contains 70% composted pine bark (0–5 mm) and 30% coco peat with 2 pellets of yates osmocote slow-release fertiliser positioned in the bottom 3 cm of pot. Pots were watered with 20 mL of tap water and left in a tray with clear plastic cover and placed inside a growth cabinet set at 25 ± 1 °C with a 16 h photoperiod and 78% humidity for acclimatization. Plants were regularly assessed for evidence of effective acclimatization, signified by new growth.

### Selenium dosing and elemental analysis of plant tissues

**Fig. 2:**
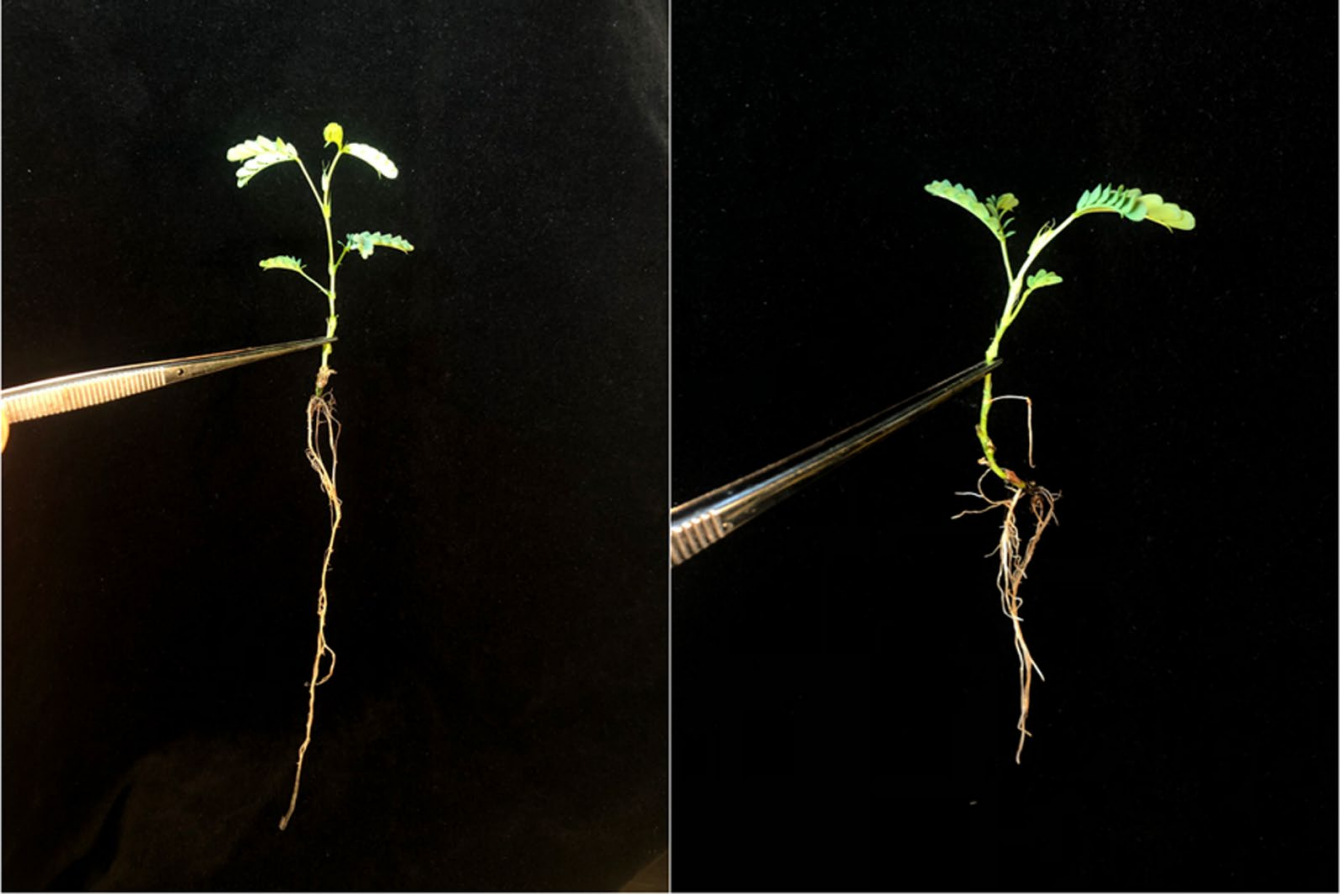
Se dosed clonal plantlets after being removed from soil after 4–5 weeks and washed prior to ICP-AES analysis. Well-developed root with root branching can be seen

To assess Se accumulation of plantlets after micropropagation, acclimatized clones were watered with a Se solution. Pots were dosed with 30 mL water (control), or with a solution containing both Na_2_SeO_4_ and NaSeO_3_ to achieve 100 mg kg^−1^ Se at a ratio of 50:50 selenate to selenite. A petri dish was placed under each pot to catch excess Se solution. Four weeks after dosing plantlets were removed from soil and thoroughly washed to remove any soil (Fig. 2). Using scissors plants were cut at the base of the shoot and had roots and shoots placed into separate envelopes. Harvested plant tissue samples were dried in a drying oven at 60 °C for 70 h. Samples were manually broken up and weighed into 6 mL polypropylene tubes. Samples were pre-digested using 2 mL HNO_3_ (70% v/v) for 40 h before being digested in a block heater (Thermo Scientific™ digital dry bath) for a 2-h programme (1 h at 70 °C followed by 1 h at 125 °C), filtered and diluted to 10 ml with ultrapure water (Millipore 18.2 MΩ·cm at 25 °C) before analysis with inductively coupled plasma atomic emission spectroscopy (ICP-AES) with a Thermo Scientific iCAP 7400 instrument for macro-elements (Na, Mg, Al, P, S, K, Ca), trace-elements (Cr, Mn, Fe, Co, Ni, Cu, Zn) and ultra-trace elements (As, Se, Cd, Tl).

### Statistical analysis

Statistical analysis was conducted with Graphpad Prism 9 software to determine statistical significance between treatment groups at the 0.05 level. Various statical test were used depending on the data type; on parametric data comparing more than two treatments, analysis of variance was used followed by Tukey’s Honest Significant Difference post hoc tests; on parametric data comparing responses of only two treatments, two tailed t test were used and on non-parametric data, Chi squared test of independence was used for larger data sets and Fisher’s exact test for smaller sample sizes. If a significant difference was found in the trial, pair-wise comparisons were carried out.

## Results and discussion

### Selenium media concentration effect on seed germination and nodal vigour

**Fig. 3:**
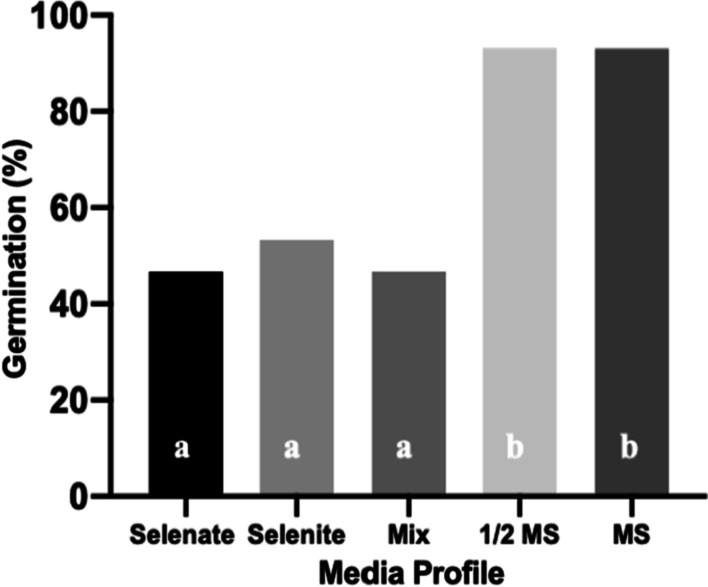
Percent germination of sterilised *Neptunia amplexicaulis* seeds cultured on various media profiles; Full strength MS with addition of 40 ppm Se in the form of selenate, selenite, or a mix of both at a ratio of 20:20, half strength MS and plain full strength MS. Columns with same letter indicate no significant difference at P < 0.05. (n ≤ 15 per treatment)

The endemic nature of *N. amplexicaulis* to the Se-rich soils surrounding the Richmond area suggests an intrinsic Se requirement of the species. Additionally, seeds of this species have been recorded containing up to 500 µg Se g^−1^, suggesting Se may play an important role in early seedling development and/or defence [5]. Although soil in the Richmond has been recorded with up to and above 40 mg kg^−1^ Se [6], this trial suggested that 40 mg kg^−1^ Se negatively affected both germination success and growth of seedlings in in vitro conditions. Seeds sown in media containing Se showed a near 50% reduction in germination rate when compared to seeds sown in plain MS [Fig. 3, X^2^ (1, N = 75) = 14.0625, *p* ≤ 0.001]. Those seeds that did germinate in the Se amended media showed notable reduction in plant vigour, displaying signs of yellowing and leaf drop, and eventually died after 3–4 weeks. There was no difference in germination rate or growth of seedlings on full-strength MS versus half MS in a 1-month period. All nodes exposed to media containing 20 mg kg^−1^ Se (selenate, selenite, and a mix of both) displayed browning of the basal end and died within 2 weeks of culture. Selenium was therefore not included in media of future multiplication trials. This experiment suggests environmental Se is not essential for early stages of in vitro *N. amplexicaulis* seedling or nodal growth. Further study could be directed towards investigating the response of *N. amplexicaulis* to various concentrations and compounds of Se in media, possibly developing a rate, form and timing of Se delivery that would benefit plant vigour.

### Effect of growth regulators on shoot multiplication of seedling nodes

Nodal explants showed elongation of axillary buds within 8–14 days of transfer onto media regardless of inclusion or concentration of PGRs. Differences in shoot multiplication in terms of both the mean number of shoots produced per node and the percentage of nodes with multiple shoots showed a positive effect of BA at concentrations tested under 3.0 mg L^−1^. Inclusion of NAA in media in conjunction with BA was seen to increase shoot multiplication compared to trials with BA alone however ANOVA post hoc test revealed differences were not significant at 0.05 level (Table 1). Both the highest rate of shoot multiplication per node (2.1) and maximum percentage of nodes displaying multiple shoots (53.45%) were observed in cultures supplemented with 2.0 BA mg L^−1^ + NAA 0.2 mg L^−1^. Similar concentrations were observed to be optimal in studies of other related species *Desmodium gangeticum* and *Stanleya pinnata* [21, 22]. Concentration of BA at 3 mg L^−1^showed a drastic reduction in shoot multiplication likely due to heavy callusing at the basal end of the node. In general, addition of BA and the increase in shoot number was correlated with significantly reduced individual shoot length when compared to plain MS media, where fewer but longer shoots were seen. Many additional axillary buds and shoots beginning to elongate were observed on the majority of the BA-treated nodal explants, however as these were under 5 mm, they were not recorded in multiplication rate calculations. It is possible that many of these buds and small shoots would have elongated to a length over 5 mm and increased the multiplication levels with further subculturing cycles, as seen for the micropropagation of related species *Clitoria ternatea* and *Desmodium gangeticum* [17, 21].

**Table 1:** Shoot growth and multiplication of nodal explants from in vitro grown *Neptunia amplexicaulis* seedlings cultured on full strength MS media supplemented with varying concentrations and combinations of BA and NAA

BA (mg/L)	NAA (mg/L)	Percent of explants with multiple shoots	No. shoots per node	Length of tallest shoot (mm)
0	0	0	0.96 ± 0.02^bc^	45.14 ± 6.18^a^
1.0	0	50 ± 11.8^a^	1.5 ± 0.23^abc^	30.4 ± 5.81^ab^
2.0	0	23.68 ± 6.9^b^	1.44 ± 0.24^a^^bc^	22.4 ± 2.46^ab^
3.0	0	16.7 ± 7.6^b^	0.58 ± 0.1^c^	11.25 ± 2.5^b^
1.0	0.1	48.15 ± 9.6^a^	1.54 ± 0.16^ab^	20.57 ± 2.9^b^
2.0	0.2	53.45 ± 6.5^a^	2.1 ± 0.16^a^	19.82 ± 1.8^b^
3.0	0.3	11.53 ± 6.3^b^	0.9 ± 0.21^bc^	14.83 ± 3.26^b^
		n ≤ 58	n ≤ 58	n ≤ 21

Values are means ± SE after 4 weeks on media. Means followed by the same letter in a column indicate no significant difference by Tukey’s multiple comparison test or pairwise Fisher's exact test (P < 0.05)

Variation in multiplication rates was noted between cultures derived from different seedling mother plants, e.g., explants from one mother plant displayed an average 4.7 shoots per node whereas those from another in the same treatment showed < 1 shoot per node. As the population of *Neptunia* seedlings used were derived from outcrossed seed, this indicates a genotypic influence on shoot multiplication or general amenability to PGR exposure. This highlights the relevance of genotype not only potentially to Se uptake characteristics, but to culture performance, and suggests that genotype-specific optimisations using mature nodes of known high-SE-accumulating genotypes may be required in future. This phenomenon has been observed in related studies where developed tissue culture protocols display genotypic-dependent responses [23, 24].

### Effect of growth regulators on rooting of micro-shoots

Micro-shoots excised from nodal explants of *N. amplexicaulis* displayed signs of rooting between 7 and 28 days from initiation onto rooting media. Rooting was shown to occur at some level across all media compositions. In general, the addition of NAA to rooting media improved root induction, with highest percentage of rooting (30.78%) seen for 0.2 mg L^−1^ NAA, which decreased with increasing concentration of NAA. Chi squared test of independence however showed no significant difference in root induction between any rooting treatments [X^2^ (3, N = 129) = 5.9295, *P* = 0.12]. Root length was significantly decreased with addition of NAA compared to plain MS, however no significant difference was seen between NAA treatments. Root number was not significantly different between any treatment (Table 2).

**Table 2:** Rooting response of in vitro generated *N. amplexicaulis* shoots cultured on full strength MS supplemented with varying concentrations of NAA

NAA (mg/L)	Percent of shoots with roots	No. roots per shoot	Root length (mm)
0	14.29 ± 6.6^a^	2 ± 0.41^a^	40.25 ± 5.27^a^
0.2	30.78 ± 7.4^a^	2.75 ± 0.33^a^	21.45 ± 4.88^b^
0.6	23.5 ± 7.2^a^	2.16 ± 0.52^a^	12.5 ± 1.6^b^
1.0	14.7 ± 6.1^a^	2.17 ± 0.48^a^	14.62 ± 2.46^b^
	n ≤ 39	n ≤ 12	n ≤ 33

Values are means ± SE after 4 weeks on media. Means followed by the same letter in a column indicate no significant difference by Tukey’s multiple comparison test or pairwise Fisher's exact test (P < 0.05)

Studies on related Fabaceae species generally reported greater efficacy of the rooting protocols than what was experienced in this study. Employing a greater diversity of rooting media profiles such as supplementing NAA with other auxins (e.g., IBA or IAA) or utilising combinations of auxins may have provided an improved rate of rooting. Related species studies on *Desmodium gangeticum* [21] and *Clitoria ternatea* [17] reported the use of shoots over 20 mm in length in rooting trials, it is possible the use of longer shoots, or shoots derived from additional subculture cycles, in this study may have resulted in greater rooting response.

### Acclimatization

**Fig. 4:**
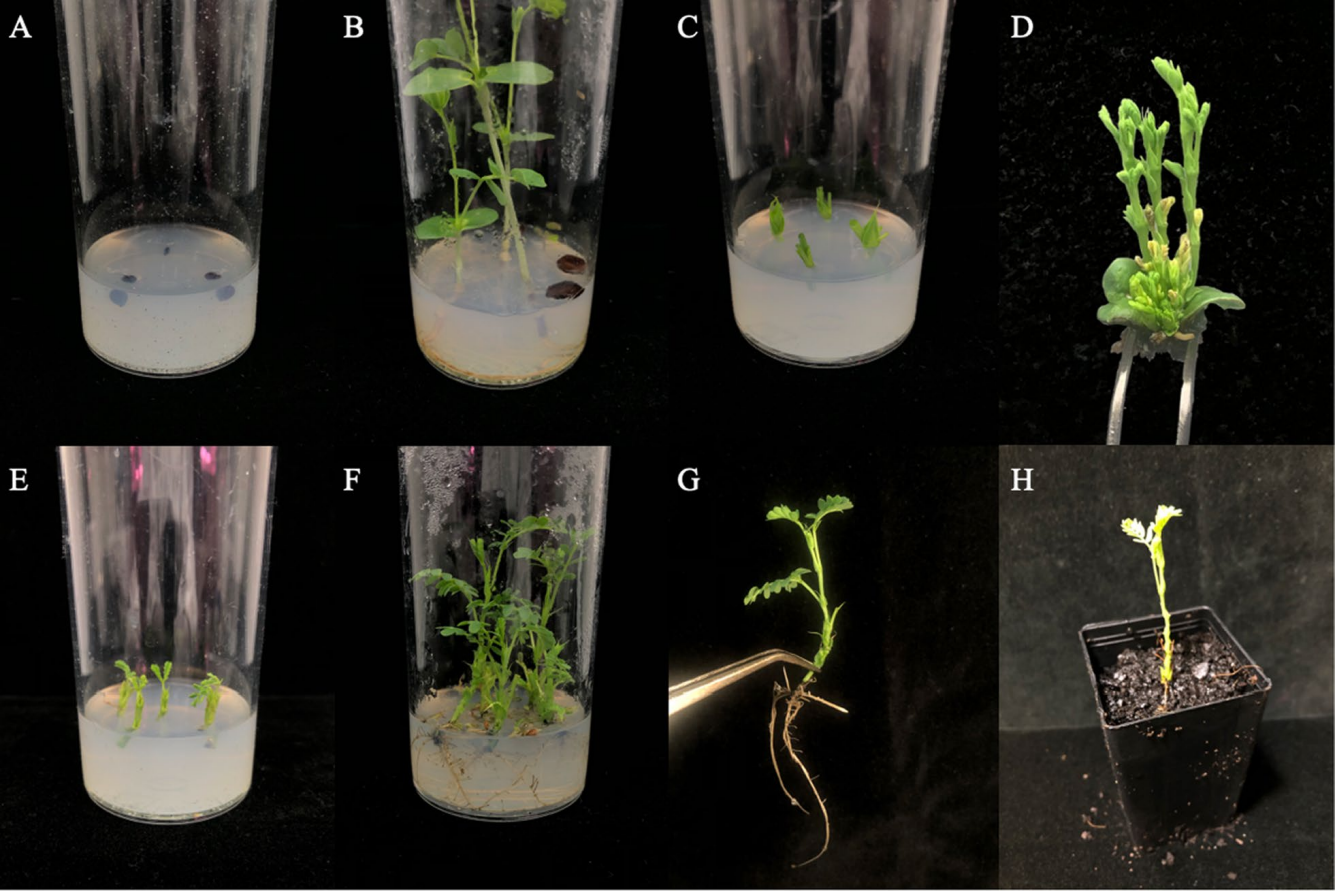
Images of the various distinct stages in *Neptunia amplexicaulis* nodal micropropagation. **A** Seeds in media for germination, **B** seedlings after 4 weeks of growth, **C** Nodes from 4-week-old seedlings in multiplication media, **D**. Node displaying multiple shoots, **E** Elongated shoots in rooting media, **F** Rooted shoots in media, **G** Rooted shoot removed from media, **H** Acclimatised plantlet

Acclimatization resulted in 95% survival of in vitro rooted shoots after 4 weeks. Leaf yellowing and dropping occurred shortly after shoots were transplanted to soil substrate however a healthy green appearance resumed after 1–2 weeks. This likely occurred as roots developed further and became accustomed to the reduced water and nutrient availability of the soil substrate compared to culturing media [25, 26]. Overall, the high rate of acclimatization indicates the roots produced through this micropropagation protocol were functional and permitted survival of plantlets in an ex vitro soil environment (Fig. 4).

### Plantlet Se accumulation assessment

**Fig. 5:**
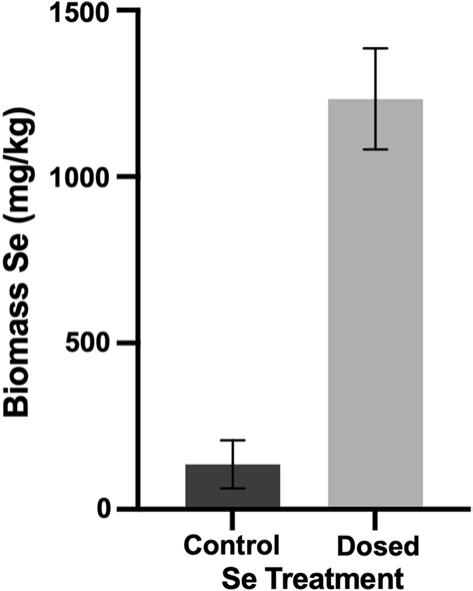
Selenium biomass concentration for clonal propagated control plants and plants grown in soil spiked to 100 ppm Se with selenate and selenite at a ratio of 50:50. Two tailed t test showed a significant difference at P < 0.05 level. (*P* ≤ 0.001, n ≤ 9)

**Fig. 6:**
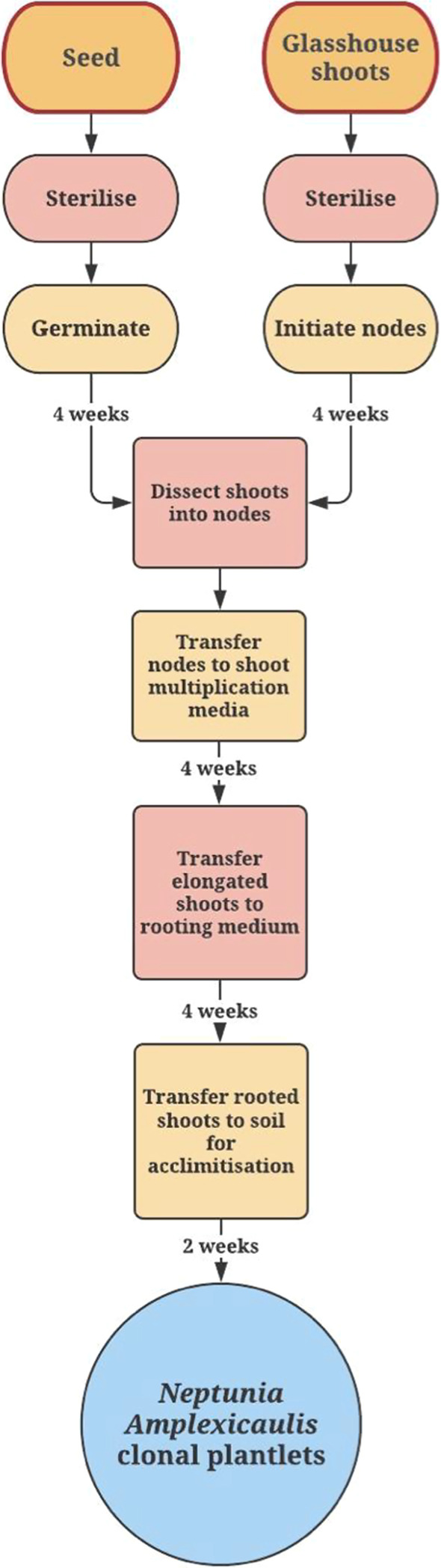
Flow chart presenting main steps for the nodal culture micropropagation of *N. amplexicaulis.* Micropropagation from seed shown on the left side and mature nodes are on the right. Steps in which the process is the same for both types of plant material are in the middle of the diagram

The ICP-AES results for Se tissue concentration suggested that the clonal plantlets produced through the seedling nodal culture micropropagation protocol retained their ability to hyperaccumulate Se to a high degree. The average concentration of Se for the 100 mg Se kg^−1^ Se dosed plantlets was 1233 µg Se g^−1^, compared to the control groups which had an average concentration of 135 µg Se g^−1^ (Figs. 5, 6*P* =  < 0.001). The levels of Se in plantlets would likely increase with prolonged exposure to dosed soils for a period longer than four weeks, likewise concentrations in control plantlets would drop with increased biomass.

The coefficient of variation within clonal groups was relatively low, ranging from 8.76 to 42.32% for Se tissue concentration and 12.67–23.59% for biomass dry weight. Variation between the clonal groups was also relatively low, with a coefficient of variation of 36.91% and 42.89% for Se tissue concentration and biomass dry weight respectively. This contrasted greatly with the huge degree of variation seen in a study conducted by Harvey et al. which looked at the distribution and concentration of Se in 2-month-old, seed germinated *N. amplexicaulis* plants grown in sandy soil. The study reported ranges of Se concentration between 1380 and 13,600 mg kg^−1^ in young leaf tissue a near tenfold variation. The low variation within clonal groups seen in the micropropagated plants can be attributed to the shared genetic material between clones. Low variation between clonal groups is likely a result of the uniform conditions experienced by the clones and the young age of the plantlets. As plants are grown within a controlled and consistent environment, reducing environmental effects which may impact variation in plant growth, this suggests this novel micropropagation protocol can produce clonal plants which display low variation in Se tissue concentration and biomass generation both within and between clonal groups, ideal for phytoextraction applications and yield consistency.

### Potential applications

Micropropagated populations of *N. amplexicaulis* could be employed in phytoextraction programs in areas of elevated soil Se concentration as a means of phytoremediation to reduce Se levels and mitigate instances of Se toxicity to cattle and local populations. Alternatively, biomass of *N. amplexicaulis* could be harvested and processed to provide a natural source of bioavailable Se compounds for use in pharmaceutical supplement products or have foliage applied to agricultural fields for soil Se bio-fortification. Clonal produced plantlets are ideal for these applications due to the ability to generate clones of individuals screened for superior Se hyperaccumulation characteristics, improving accumulation efficiency while decreasing accumulation variation, as seen in other industries which have similar goals maintaining consistency [11, 12, 27]. These clones also have significant potential to be utilised in Se hyperaccumulation studies, such as genetic and gene expression projects or other in vitro studies.

## Conclusions

This study describes the first ever protocol for the in vitro germination and nodal culture micropropagation of *N. amplexicaulis*, generating clonal plantlets, which retained the ability to hyperaccumulate Se to uniform levels. *N. amplexicaulis* showed high amenability to in vitro growth conditions as has been reported for other related Fabaceae species. It is likely that rates of shoot multiplication and rooting could be further optimised by prolonging the shoot multiplication phase and exploration of additional shoot multiplication and rooting techniques and media profiles. Regardless, this protocol provides a proof-of-concept for micropropagation of Se hyperaccumulating clonal plantlets with potential for industrial phytoextraction applications and future research objectives benefiting the environment and human health.
